# Viral Hepatitis E Outbreaks in Refugees and Internally Displaced Populations, sub-Saharan Africa, 2010–2020

**DOI:** 10.3201/eid2805.212546

**Published:** 2022-05

**Authors:** Angel N. Desai, Amir M. Mohareb, Mubarak Mustafa Elkarsany, Hailemichael Desalegn, Lawrence C. Madoff, Britta Lassmann

**Affiliations:** University of California–Davis, Sacramento, California, USA (A.N. Desai);; International Society for Infectious Diseases, Brookline, Massachusetts, USA (A.N. Desai, M.M. Elkarsany, L.C. Madoff, B. Lassmann);; Massachusetts General Hospital, Boston, Massachusetts, USA (A.M. Mohareb);; Karary University, Khartoum, Sudan (M.M. Elkarsany);; St. Paul’s Hospital MMC, Addis Ababa, Ethiopia (H.D. Desalegn);; University of Massachusetts Medical School, Worcester, Massachusetts, USA (L.C. Madoff)

**Keywords:** hepatitis, hepatitis E, HEV, refugees, ProMED, ProMED-mail, displaced persons, outbreaks, viruses, sub-Saharan Africa, Africa, Chad, Niger, Sudan, South Sudan, Namibia, Nigeria, Burkina Faso

## Abstract

Hepatitis E virus is a common cause of acute viral hepatitis. We analyzed reports of hepatitis E outbreaks among forcibly displaced populations in sub-Saharan Africa during 2010–2020. Twelve independent outbreaks occurred, and >30,000 cases were reported. Transmission was attributed to poor sanitation and overcrowding.

Hepatitis E virus (HEV) is a common etiology of acute viral hepatitis worldwide (*1*). Large-scale, often protracted outbreaks caused by HEV infection in refugee and internally displaced person (IDP) settlements and camps have occurred (*1*), particularly in sub-Saharan Africa, a region with nearly one third of the global forcibly displaced population (*2*). Previous epidemiologic studies of HEV infections in forcibly displaced persons have focused on singular events (*3*,*4*). The objective of this study was to identify trends in HEV outbreaks among forcibly displaced populations in sub-Saharan Africa.

We conducted a focused review of all English-language curated reports posted on ProMED-mail (ProMED) during 2010–2020 concerning HEV in forcibly displaced populations in sub-Saharan Africa. ProMED uses formal and informal disease surveillance mechanisms to rapidly report emerging disease events in animals, humans, and plants globally (*5*). It has been validated as a rapid and accurate tool for determining and describing global outbreaks. We verified all reports via PubMed, ReliefWeb, the UN High Commission for Refugees, World Health Organization (WHO), and references secondarily collected from ProMED. We used the keyword “hepatitis E” in applicable search engines for reports published during 2010–2020. We included records documenting “refugee(s) and/or asylum seeker(s) and/or internally displaced person(s)” in sub-Saharan Africa as defined by the World Bank (*6*). We considered outbreaks unique on the basis of date and location of cases. When screening ProMED reports, we used the most recent report pertaining to an outbreak. In cases where discrepancies existed between data sources reporting on the same outbreak, we retained the higher number of case counts. Three independent investigators (A.D., B.L., and A.M.) manually reviewed the databases.

Twelve hepatitis E outbreaks among forcibly displaced persons resulting in a total of >30,000 suspected or confirmed cases of acute HEV and >610 deaths were reported during 2010–2020 (Appendix Table). Outbreaks occurred in Sudan, South Sudan, Ethiopia, Chad, Niger, Namibia, Burkina Faso, Kenya, and Nigeria (Figure). One outbreak in displaced persons in South Sudan’s Bentiu camp for internally displaced persons that included >1,000 cases since 2019 was not included in this analysis because it continued beyond 2020. The largest outbreak of acute HEV infections (>11,000 cases) was reported in a protracted outbreak in the Upper Nile, South Sudan, during July 2012–October 2013, among persons fleeing violence in Sudan in 2011. The most common contributors to hepatitis E outbreaks reported were overcrowding, poor sanitation, and flooding.

**Figure:**
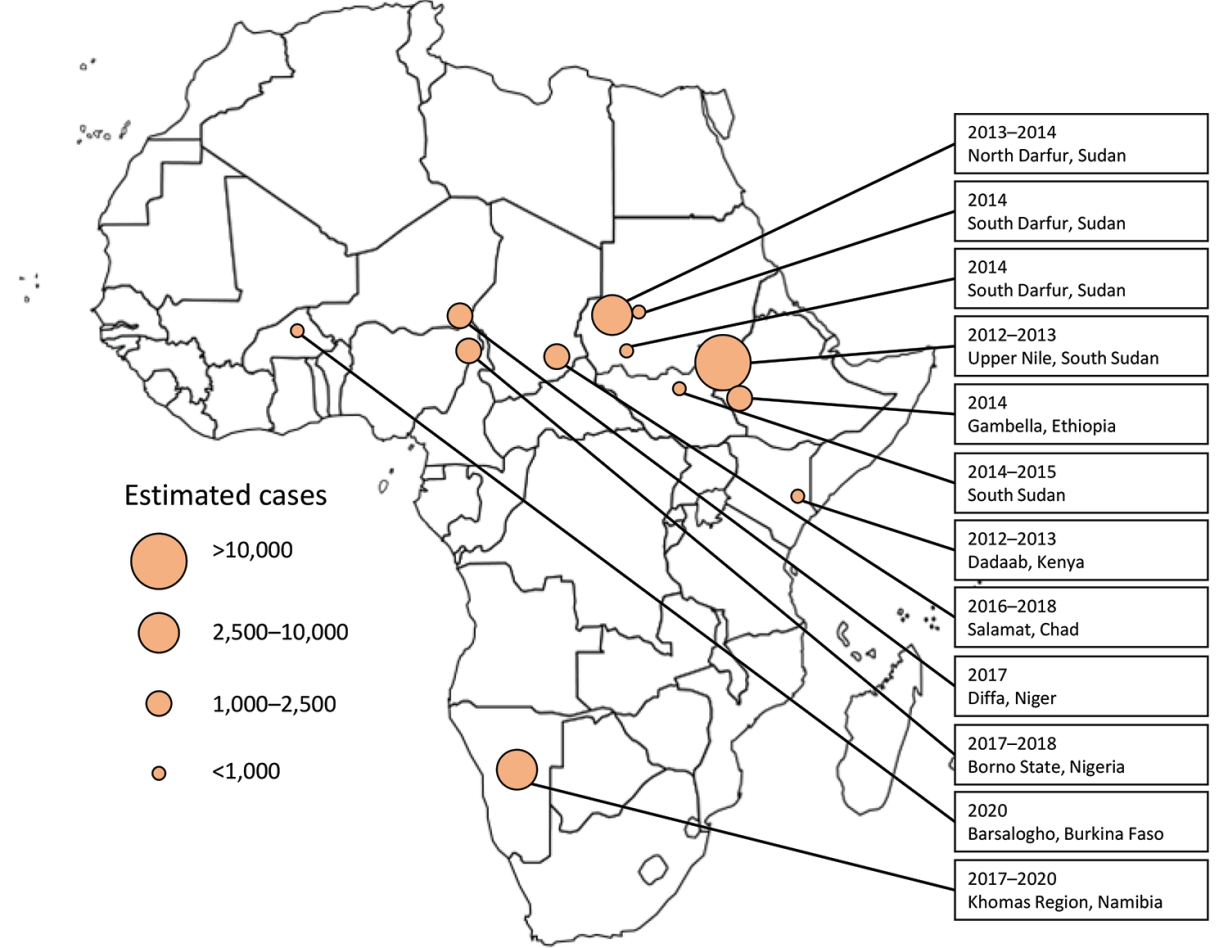
Geographic distribution of acute hepatitis E virus outbreaks reported among displaced persons in sub-Saharan Africa, 2010–2020.

Prior studies have demonstrated the proclivity of HEV transmission in settings such as refugee and IDP camps; close quarters, inadequate sanitation and hygiene, and the constant introduction of new, susceptible persons into camps provided the conditions necessary for forward transmission (*3*,*4*). We could not calculate accurate case-fatality rates given the uncertainty surrounding the total number of true cases and deaths reported. Population-based studies during disease outbreaks of hepatitis E have placed mortality rates at 0.07%–0.6%; we noted substantial variability particularly for high-risk populations such as pregnant women (*1*). Cases and fatalities in pregnant women were reported for 3 hepatitis E outbreaks in this series: 2 reported deaths among 18 cases in pregnant women in Ethiopia (2014); 17 reported deaths in pregnant women in Niger (2017), comprising 45% of the recorded deaths in that outbreak; and 12 reported deaths in pregnant women in Namibia (2019).

The first limitation of this study is that case definitions may vary between settings, and confirmatory testing was not always reported. Second, mild and asymptomatic cases are often unreported, and the relatively long incubation period for HEV infection may hinder diagnosis and reporting. Third, misclassification bias is possible, especially because many of the settings are endemic for other causes of acute jaundice syndrome, such as malaria and yellow fever, and diagnostic testing was infrequent. Those factors also limited our ability to conduct a pooled analysis on the data. 

Despite these limitations, this study demonstrates the high potential for HEV to cause outbreaks in communities with recently displaced persons. Of note, all of the reported outbreaks in this study occurred in the context of highly crowded camps or settlements, supporting the association between hepatitis E outbreaks and those environments. Given that some of the outbreaks noted in this analysis appeared to cross national borders, genetic sequencing to validate related strains may be useful for disease surveillance and prevention efforts. Additional data are needed to evaluate the potential utility of HEV vaccination in outbreaks and the barriers to vaccinating residents of refugee and IDP settlements. Water, sanitation, and hygiene measures are critical to reducing disease outbreaks, as is improved cross-border communication to prevent and manage future outbreaks. Clinicians and relief staff working with displaced populations should be vigilant for signs of hepatitis E disease, particularly among high-risk hosts such as pregnant women. Resources must be devoted to improving HEV surveillance, diagnostic capabilities, and response efforts for refugee and displaced populations.

Appendix Additional information about viral hepatitis E outbreaks in refugee and displaced-person populations in sub-Saharan Africa, 2010–2020.
