# Supplementary material for: Viral Hepatitis E Outbreaks in Refugees and Internally Displaced Populations, sub-Saharan Africa, 2010–2020
Source: Emerg Infect Dis. 2022 May;28(5):1074–6. doi: 10.3201/eid2805.212546 (PMC9045430; doi:10.3201/eid2805.212546)
Supplement: Appendix — Additional information about viral hepatitis E outbreaks in refugee and displaced-person populations in sub-Saharan Africa, 2010–2020. [file 21-2546-Techapp-s1.pdf]

Article DOI: https://doi.org/10.3201/eid2805.212546

Viral Hepatitis E Outbreaks in Displaced Populations, Sub-Saharan Africa, 2010–2020

Appendix

Appendix Table. Published reports for outbreaks of viral hepatitis E in sub-Saharan Africa, 2010–2020

Region and country	Site (pop.)	Year	No. suspected cases (time period)	Confirmatory testing in select cases	No. deaths*	References
Upper Nile, South Sudan	Maban County (110,000)	2012–2013	>11,000 (Jul 2012–Oct 2013)	Yes, PCR and rapid antibody testing	241	CDC (*3*)
						ProMed (*12*)
Garissa County, Kenya	Dadaab (460,000)	2012–2013	339 (Jul–Nov 2012)	Yes, PCR and ELISA antibody testing	10	Ahmed et al. (*4*)
North Darfur, Sudan	El Sareif (59,000)	2013–2014	2,572 (Jan 2013–Feb 2014)	Not available	34	ProMed (*13*)
South Darfur, Sudan	Nyala (90,000)	2014	628 (Jan–Sep 2014)	Yes, testing modality not specified	150	OCHA (*5*)
						ProMed (*14*)
Gambela, Ethiopia	Leichuor, Kule, Tierkidi (143,000)	2014	1,117 (Apr 2014–Jan 2015)	Yes, PCR and rapid antibody testing	21	Browne et al. (*1*)
						ProMed (*15*)
South Sudan	Bentiu, Mingkamen, Lankien (200,000)	2014–2015	729 (Aug 2014–Aug 2015)	Yes, PCR and ELISA antibody testing	4	WHO (*6*)
						ProMed (*16*)
North Darfur, Sudan	Sortony (21,000)	2016	134 (May–Jul 2016)	Yes (testing modality not specified)	Not reported	OCHA (*7*)
						ProMed (*17*)
Salamat, Chad	Am Timan, Aboudeia, Amsinéné, Mouraye, Foulonga	2016–2018	1,874 (Aug 2016–Jan 2018)	Yes (testing modality not specified)	23	WHO Africa Regional Office (*8*)
						ProMed (*18*)
Lake Chad Basin, Niger	Diffa (240,000)	2017	1,917 (Jan–Sep 2017)	Yes (PCR and ELISA antibody testing)	38	Lagare et al. (*2*)
						ProMed (*19*)
Lake Chad Basin, Nigeria	Borno State (1.4 million)	2017–2018	1,815 (Feb 2017–Feb 2018)	Yes (ELISA antibody testing)	8	Nigeria Centre for Disease Control (*10*)
						ProMed (*20*)
Namibia	Windhoek, Khomas region†	2017–2020	7,457 (Oct 2017–Mar 2020)	Yes (antibody testing)	65	WHO Africa Regional Office (*9*)
						ProMed (*21*)
North Central Region, Burkina Faso	Barsalogho District (99,000)	2020	442 (Jul–Nov 2020)	Yes (PCR)	16	WHO (*11*)

Region and country	Site (pop.)	Year	No. suspected cases (time period)	Confirmatory testing in select cases	No. deaths*	References
						ProMed (*22*)

*Totals include suspected and confirmed deaths.
†Most cases were reported from informal settlements within the capital district Windhoek, though by April 2018 the outbreak involved other surrounding regions as well. The cases reported here are a summary of the entire series of outbreaks.

References

1. Browne LB, Menkir Z, Kahi V, Maina G, Asnakew S, Tubman M, et al.; Centers for Disease Control and Prevention (CDC). Notes from the field: hepatitis E outbreak among refugees from South Sudan—Gambella, Ethiopia, April 2014–January 2015. MMWR Morb Mortal Wkly Rep. 2015;64:537. PubMed

2. Lagare A, Ibrahim A, Ousmane S, Issaka B, Zaneidou M, Kadadé G, et al. Outbreak of hepatitis E virus infection in displaced persons camps in Diffa Region, Niger, 2017. Am J Trop Med Hyg. 2018;99:1055–7. PubMed https://doi.org/10.4269/ajtmh.17-0950

3. Centers for Disease Control and Prevention (CDC). Investigation of hepatitis E outbreak among refugees—Upper Nile, South Sudan, 2012–2013. MMWR Morb Mortal Wkly Rep. 2013;62:581–6. PubMed

4. Ahmed JA, Moturi E, Spiegel P, Schilperoord M, Burton W, Kassim NH, et al. Hepatitis E outbreak, Dadaab refugee camp, Kenya, 2012. Emerg Infect Dis. 2013;19:1010–2. PubMed https://doi.org/10.3201/eid1906.130275

5. OCHA; ReliefWeb. Hepatitis outbreak kills 150 people in South Darfur. 3 Sep 2014. [cited 2021 Dec 2]. https://reliefweb.int/report/sudan/hepatitis-outbreak-kills-150-people-south-darfur-s-kalma-idp-camp

6. WHO Early Warning and Response Network and integrated disease surveillance and response (South Sudan). Geneva: The Organization; 2015.

7. OCHA. Sudan: humanitarian bulletin. 31 July 2016 [cited 2021 Dec 2]. https://reliefweb.int/report/sudan/sudan-humanitarian-bulletin-issue-31-25-31-july-2016-enar

8. World Health Organization. WHO Africa Regional Office outbreak report. March 2018 [cited 2021 Dec 2]. https://reliefweb.int/sites/reliefweb.int/files/resources/OEW9-24022032018.pdf

9. World Health Organization. WHO Africa Regional Office outbreak report. August 2019. [cited 2021 Dec 8]. https://www.afro.who.int/publications/outbreak-hepatitis-e-virus-hev-namibia-situation-report-sitrep-no-64

10. Nigeria Centre for Disease Control. Weekly epidemiological report. 28 July 2017 [cited 2021 Dec 2]. https://reliefweb.int/sites/reliefweb.int/files/resources/28_July_2017.pdf

11. WHO Disease outbreak news. November 27, 2020. 2020 [cited 2021 Dec 7]. https://www.who.int/emergencies/disease-outbreak-news/item/2020-DON300

12. ProMED. Hepatitis E—South Sudan (02): refugee camps, fatalities. ProMed. 2013 Feb 15 [cited 2022 Jan 1]. http://www.promedmail.org, archive no. 20130216.1545704. http://www.promedmail.org/post/1545704

13. ProMED. Hepatitis E—Sudan: (North Darfur). ProMed. 2013 May 23 [cited 2022 Jan 1]. http://www.promedmail.org, archive no.20130524.1735825. http://www.promedmail.org/post/1735825

14. ProMED/MENA. Hepatitis E—Sudan (02): (South Darfur, Blue Nile) displaced persons. ProMed. 2014 Sep 25 [cited 2022 Jan 1]. http://www.promedmail.org, archive no.20141002.2820794. https://promedmail.org/promed-post/?place=2820794,96#promedmailmap

15. ProMED. Hepatitis E—Ethiopia: (Gambella): refugees. ProMed. 2014 Aug 8 [cited 2022 Jan 1]. http://www.promedmail.org, archive no. 20140810.2676571. https://promedmail.org/promed-post/?place=2676571,32385

16. ProMED. Hepatitis E—South Sudan: (Lakes): displaced persons camp. ProMed. 2014 Sep 7 [cited 2022 Jan 1]. http://www.promedmail.org, archive no. 20140908.2757656. https://promedmail.org/promed-post/?place=2757656,8402

17. ProMED. Hepatitis E—Sudan (North Darfur) internally displaced persons camp. ProMed. 2016 Aug 9 [cited 2022 Jan 1]. http://www.promedmail.org, archive no. 20160810.4405317. http://promedmail.org/direct.php?id=20160810.4405317

18. ProMED. Hepatitis E—Chad (03): (Salamat). ProMed. 2017 Feb 25 [cited 2022 Jan 1]. http://www.promedmail.org, archive no. 20170226.4866244. https://promedmail.org/promed-post/?place=4866244,57#promedmailmap

19. ProMED. Hepatitis E—Africa: (Lake Chad Basin). ProMed. 2017 Aug 10 [cited 2022 Jan 1]. http://www.promedmail.org, archive no. 20170810.5241448. https://promedmail.org/promed-post/?place=5241448,20388.

20. ProMED. Hepatitis E—Nigeria (03): (Borno) internally displaced persons camp. ProMed. 2017 Aug 11 [cited 2022 Jan 1]. http://www.promedmail.org, archive no. 20170814.5244528. https://promedmail.org/promed-post/?place=5244528,621#promedmailmap

21. ProMED. Hepatitis E—Namibia (Windhoek). ProMed. 2017 Dec 20 [cited 2022 Jan 1]. http://www.promedmail.org, archive no.20171225.5522167. https://promedmail.org/promed-post/?place=5522167,3585#promedmailmap

22. ProMED. Hepatitis E—Burkina Faso. ProMed. 2020 Nov 27 [cited 2022 Jan 1]. http://www.promedmail.org, archive no. 20201202.7988324. https://promedmail.org/promed-post/?place=7988324,49#promedmailmap
